# Bacteriological assessment of stethoscopes used by healthcare workers in a tertiary care centre of Nepal

**DOI:** 10.1186/s13104-017-2677-7

**Published:** 2017-07-28

**Authors:** Sangita Thapa, Lokendra Bahadur Sapkota

**Affiliations:** 1 Department of Clinical Microbiology & Immunology, Chitwan Medical College & Teaching Hospital (CMCTH), Bharatpur-10, Chitwan, Nepal; 2 Department of Biochemistry, Chitwan Medical College, Bharatpur-10, Chitwan, Nepal

**Keywords:** Contamination, Health care workers, Health care associated infections, Stethoscope

## Abstract

**Objective:**

Stethoscope is a medical device universally used by health care workers. Stethoscope may transmit pathogens among patients and health care workers if it is not disinfected. The objective of this study was to, determine the level of stethoscope contamination used by health care workers, survey the practices of disinfecting the stethoscope, identify various microorganisms and assess their role as potential pathogens and determine the effectiveness of 70% ethanol as a disinfecting agent.

**Results:**

This was a cross-sectional study conducted in the department of Microbiology, Chitwan Medical College, Bharatpur, Nepal. Stethoscopes of 122 health care workers from different departments were included in this study. Out of a total 122 diaphragms, 88 (72.1%) were colonized. Only 71 (58.1%) bells and 152 earpieces (66.2%) were contaminated. *Micrococcus* and coagulase negative staphylococci were predominantly isolated species. The contamination was lowest among stethoscopes cleaned after touching every patient (11.5%) and the difference is statistically significant (P < 0.0001). Significantly lower level of contamination (13.6%) were found on stethoscopes cleaned everyday (P < 0.0001). Only 8.5% stethoscope showed growth with decreased number of colonies after disinfecting the stethoscopes with 70% ethanol. Thus, demonstrating the effectiveness of disinfection.

**Electronic supplementary material:**

The online version of this article (doi:10.1186/s13104-017-2677-7) contains supplementary material, which is available to authorized users.

## Introduction

The development of nosocomial infection is a significant problem in each hospital. Such infections can result due to multiple causes like development and persistence of multidrug resistant (MDR) bacteria, immunocompromised states of patients, and mechanical transmission of microorganisms [1]. Some non-critical medical devices routinely used by Health care workers (HCWs) such as stethoscopes, blood pressure cuffs electronic thermometers, latex gloves, masks, pens, and white coats play significant role in the transmission of health care associated infections (HCAIs) [2]. Among these devices, stethoscopes routinely used by HCWs pose a potential threat for the transmission of HCAIs in the hospital settings [3]. Stethoscopes frequently come in contact with many patients. During such contacts, microorganisms can colonize on the stethoscopes which could further spread to other patients if proper disinfection practices are not followed by HCWs [4, 5]. Since, routine disinfection practice of stethoscopes are not followed by HCWs, there is high risk of transmission of multidrug antibiotic resistant microorganisms in the hospital settings [6, 7]. These include methicillin-resistant staphylococci, ceftazidime-resistant *Klebsiella pneumoniae*, vancomycin-resistant enterococci, ciprofloxin-resistant *Pseudomonas aeruginosa*, gentamicin-resistant *Pseudomonas aeruginosa*, and penicillin-resistant pneumococci [8].

Despite stethoscopes being a potential vector for the transmission of HCAIs, disinfection of stethoscopes is neglected by HCWs [9]. Stethoscope swiping using alcohol pads is the current gold standard method for the disinfection of stethoscopes [10]. Medical device like stethoscopes should be evaluated for microbial colonization frequently and HCWs should be sensitized about the regular disinfection practices to control nosocomial infections [11].

## Main text

This cross-sectional study was conducted in the Department of Microbiology, Chitwan Medical College, Bharatpur, Nepal, between August 2015 and February 2016. Stethoscopes of 122 healthcare workers from different departments were included in this study.

### Methodology

Specimens were collected from the diaphragms, bells and both earpieces. Direct inoculation onto blood agar plates was done for the earpieces while swab moistened in sterile normal saline was used for the diaphragms and bells. The diaphragms, bell and both earpieces of the stethoscopes were pressed firmly and rubbed 1 cm on both sides on blood agar and MacConkey agar. The plates were incubated aerobically at 37 °C for 48 h. Microorganisms were identified by conventional phenotypic methods [12]. Antibiotic sensitivity test (ABST) of the microorganisms was performed by Kirby-Bauer disk diffusion method. In addition, randomly 35 stethoscopes were swabbed once with 70% ethanol, allowed to dry, and then sampled.

Before sample collection all the participants were given a preformed questionnaire regarding HCWs routine stethoscope disinfection practices (Additional file 1). Survey was done on frequency and methods practiced for cleaning of stethoscopes, frequency of hand washing and barriers to cleaning of stethoscopes by HCWs.

#### Statistical analysis

All the data were entered in the Microsoft Excel and analyzed by SPSS version 20. Differences between the proportions were assessed by means of Chi square analysis. P < 0.05 was said to be statistically significant. Point estimates for the primary outcome are reported with 95% confidence interval.

## Results

Stethoscopes of 122 HCWs were sampled in the study from wards 41.8%, outpatient department (OPD) 35.2% and intensive care unit (ICU) 23% which included 70 (57.3%) males and 52 (42.6%) females. The bacterial load varied, with a minimum number of 9 colonies from a stethoscope sampled from the anesthesia department and a maximum 60 colonies from surgery ward and ICU. The results of the study was based on questionnaires which were filled by HCWs reported that 96.7% were aware that stethoscopes could transfer microorganisms, while all HCWs 100% were aware that disinfection of stethoscopes is needed. Majority of the HCWs 108 (88.5%) used stethoscope after removing the clothes of patients, while 14 (11.4%) used stethoscope without removing the clothes. Among, 122 diaphragms, 88 (72.1%) were colonized. Only 71 (58.1%) bells and 152 (66.2%) earpieces out of total 244 earpieces (122 left and 122 right) were contaminated. There were altogether 178 isolates from 88 contaminated diaphragms. *Micrococcus* and CONS were most commonly isolated (Table 1).

**Table 1:** Organisms isolated from bell, diaphragm and earpieces of stethoscope

Organisms	Number of isolates			
	Bell	Diaphragm	Earpiece (left)	Earpiece (right)
*Micrococcus* species	56	64	18	20
Coagulase negative staphylococci	16	45	11	14
Bacillus species	18	22	24	21
Diptheroids	4	6	–	–
*Staphylococcus aureus* (MSSA)	6	15	–	4
*Staphylococcus aureus* (MRSA)	10	11	–	–
*Pseudomonas* species	–	6	–	–
*Enterobacter* species	–	4	–	–
*Escherichia coli*	–	3	–	–
*Candida* species	–	2	–	–
Total no of isolates	110	178	53	59
No growth on stethoscopes	51	34	48	44
Growth on stethoscopes	71 (58.1%)	88 (72.1%)	74 (60.6%)	78 (63.9%)

– not isolated

Table 2 depicts that overall, 88 (72.1%) HCWs reported cleaning of stethoscopes by one method or the other, but 34 (27.8%) had never cleaned their stethoscopes. Out of 122 stethoscope, 22 (18%) HCWs reported regular disinfection of stethoscope after use. On comparing frequency of stethoscope disinfection practices among HCWs highest colonization was found among stethoscopes which was never cleaned 32 (94.1%) and lowest colonization among stethoscopes cleaned everyday 3 (13.6%). This was statistically significant (P < 0.0001). Highest contamination was found among stethoscopes which was not cleaned after contact with every patient 85 (88.5%) and lowest among the stethoscopes cleaned after contact with every patient 3 (11.5%) using 70% ethanol. This was statistically significant (P < 0.0001).

**Table 2:** Health care workers stethoscope disinfection practices

Methods	Number (%) of stethoscopes examined	Number (%) of stethoscopes contaminated	95% confidence interval
Frequency of disinfection of stethoscopes			
Every day	22 (18)	3 (13.6)	0.72–27.92
Alternate day	14 (11.4)	9 (64.2)	39.09–89.31
Once a week	27 (22.1)	22 (81.4)	66.72–96.08
Once a month	16 (13.1)	14 (87.5)	71.3–103.7
>Once yearly	9 (7.3)	8 (88.8)	68.2–109.4
Never cleaned	34 (27.8)	32 (94.1)	86.18–102.02
Total	122	88 (72.1)	64.14–80.06

Methods	Numbers (%)		
Methods practiced by HCW for cleaning of stethoscopes			
Methylated spirit swab	42 (34.4)	18 (42.8)	27.84–57.76
Hand sanitizer	21 (17.2)	16 (76.1)	57.86–94.34
Cloth	11 (9)	10 (90.9)	73.9–107.9
Soapy water	14 (11.4)	12 (85.7)	67.36–104.04
No agent/never cleaned	34 (27.8)	32 (94.1)	86.18–102.02
Total	122	88 (72.1)	64.14–80.06
Hand washing after each patient			
Yes	21 (17.2)	6 (28.5)	9.19–47.81
No	101 (82.7)	82 (81.1)	73.46–88.74
Total	122	88 (72.1)	64.14–80.06
Stethoscope cleaning after every patient			
Yes	26 (21.3)	3 (11.5)	0.76–23.76
No	96 (78.6)	85 (88.5)	82.12–94.88
Total	122	88 (72.1)	64.14–80.06
Barriers to cleaning of stethoscopes			
Lack of time	43 (35.2)	31 (72)	58.58–85.42
Forgetfulness/laziness	26 (21.3)	17 (65.3)	47–83.6
Lack of knowledge regarding best disinfectant	16 (13.1)	12 (75)	53.78–96.22
Lack of access to disinfectants	11 (9)	8 (72.7)	46.37–99.03
Concern for damaging one’s stethoscope	4 (3.2)	3 (75)	32.57–117.43
Sharing of stethoscopes	13 (10.6)	9 (69.2)	44.1–94.3
Unspecified	9 (7.3)	8 (88.8)	68.2–109.4
Total	122	88 (72.1)	64.14–80.06

Methylated spirit swab (63.9%) was most commonly used cleaning agent. When the methods practiced by HCWs for cleaning of stethoscopes was related to the stethoscope contamination results showed highest colonization among stethoscope which was never cleaned 32 (94.1%); lowest colonization among stethoscope which was cleaned using methylated spirit swab 18 (42.8%) and the difference is statistically significant (P < 0.0001).

The most common barrier to cleaning of stethoscope among HCWs were lack of time (35.2%), forgetfulness (21.3%), lack of knowledge regarding best disinfectant (13.1%), lack of access to disinfectants (9%), concern for damaging one’s stethoscope (3.2%), sharing of stethoscopes (10.6%) and unspecified (7.3%). Out of a total 122 HCWs, 21 (17.2%) wash their hand after touching every patient and 101 (82.7%) do not wash their hand after touching every patient. Lower bacterial contamination was found on stethoscopes of HCWs who practice hand washing after touching every patient compared to those who did not wash hands after touching every patients (28.5% vs. 81.1%; P < 0.0001). Out of 35 randomly cleaned stethoscopes, 35 (100%) showed colonization before disinfection and after disinfection only 3 (8.5%) showed colonization with decreased number of colonies (3–5), thus demonstrating the effectiveness of disinfection with 70% ethanol. This was statistically significant (P < 0.0001).

Antibiotic susceptibility testing (AST) of Gram positive bacteria showed resistance to erythromycin, cefoxitin, clindamycin, ampicillin/sulbactum and piperacillin-Tozabactum. Out of 46 *S. aureus* isolated, 21 were MRSA. Most of the isolated Gram negative bacteria showed 100% resistance to ceftriaxone, cotrimoxazole and ampicillin (Table 3).

**Table 3:** Antibiotic resistance pattern of gram positive and gram negative isolates

	E	CIP	CTR	CX	G	COT	CD	AMP	A/s	PIT	IPM	AK	VA
CONS	53.4	15.1	19.7	24.4	13.9	–	37.2	–	27.9	25.5	18.6	13.9	6.9
*S. aureus*	47.8	13	17.3	30.4	19.5	–	26	–	34.7	39.13	15.2	17.3	4.3
*P. aeruginosa*	–	33.3	16.6	–	50	100	–	100	–	–	–	00	–
Enterobacter species	–	50	00	–	25	100	–	100	–	–	–	00	–
*E. coli*	–	33.3	100	–	66.6	100	–	100	–	–	–	33.3	–

*Figures depict* %, – not tested, *P* penicillin, *E* erythromycin, *CIP* ciprofloxacin, *CTR* ceftriaxone, *C* chloramphenicol, *OX* oxacillin, *CZ* cefazolin, *G* gentamicin, *CN* cephalexin, *COT* cotrimoxazole, *AMP* ampicillin, *AZM* azithromycin, *AMC* amoxy-clavulanic acid, *AK* amikacin

## Discussion

This study showed that 72.1% stethoscopes were contaminated by different microorganisms which is similar to the contamination rates observed in previous studies by various investigators [13–15]. Health care staffs and their contaminated medical devices have been attributed as potential carriers of pathogenic microorganisms [16].

A majority 88.5% of the HCWs used stethoscopes after removing the clothes of the patients, clothes are source of a variety of microorganisms and also interferes with the conduction of sound waves. In this study, contamination of the diaphragms 72.1% was higher compared to ear piece 66.2% and bells 58.1%. This finding is comparable to the result of previous study, which reported 71–100% stethoscopes were colonized by different bacteria [17]. The diaphragm with a relatively larger flat surface area directly comes in contact with the patient. Therefore, it has higher chances of bacterial colonization and contamination. The bell due to its smaller surface area, has less chances of bacterial colonization [18]. Total 66.2% of the earpieces were contaminated. These earpieces do not play significant role in the transmission of bacteria as they lack direct contact with the patient’s skin. Bacterial colonization in the ear from the earpieces may transfer bacteria to the nose and skin leading to HCAIs.

This study shows that stethoscopes are contaminated with potentially pathogenic microorganisms. Previous study also reported that these bacteria were isolated from contaminated stethoscopes of HCWs [19]. Similarly, study conducted in this hospital revealed that these pathogenic microorganisms are most common cause of nosocomial infection [20]. Therefore, contaminated stethoscope can act as a source of nosocomial pathogens. Coagulase negative staphylococci (CONS) have the ability to acquire MDR and can be extremely virulent for population at risk. Gram negative bacilli and methicillin resistant *Staphylococcus aureus* (MRSA) were isolated from stethoscope which was a matter of concern. In this study 17.2% MRSA were isolated, which was cleaned only once a month/yearly. Other investigators reported 15.8–89% *S. aureus* on stethoscopes used by HCWs. Stethoscopes which was cleaned once a week/month gram negative bacilli were isolated. This emphasizes that there is need for regular disinfection of the stethoscopes. Other studies which isolated gram-negative bacilli reported that few of them are pathogenic [21, 22]. Similarly, most of the bacteria isolated were resistant to commonly used antibiotics. The emergence of antibiotic resistance by bacterial pathogens is a serious public health concern [23].

The contamination was lowest (11.5%) among stethoscopes cleaned after touching every patient. Previous study proved that regular disinfection of stethoscope substantially reduces transmission of bacterial pathogens [24]. The least contamination (13.6%) was found among stethoscopes cleaned everyday and highest level of contamination (94.1%) among stethoscopes which were never cleaned (P < 0.0001).

Stethoscopes disinfected using methylated spirit swab showed lower contamination (42.8%). Similarly, other study reported significant reduction in colony counts after disinfection using methylated spirit swab [25]. Lack of time (35.2%) and forgetfulness/laziness (21.3%) were common reasons for not cleaning stethoscopes, which might consequently increase HCAIs. Common habit among HCWs is hanging stethoscope around neck or carrying it outside non-patient related places such as canteen, lecture hall, meeting rooms and office etc. These practices should be avoided to prevent spread of nosocomial pathogens.

Lower contamination 28.5% was found on stethoscope of HCWs who practiced hand washing after touching each patient demonstrating importance of hand hygiene. Recently, the WHO reported that hand hygiene should be performed regularly in a effective manner which is fundamental in ensuring patient and HCWs safety [26].

This study demonstrated the effectiveness of disinfecting the stethoscopes with 70% ethanol, only (8.5%) stethoscope showed colonization with decreased number of colonies. Other study reported that regular cleaning of stethoscopes with alcohol 70% ethanol results in significant decline in the number of colony-forming units (CFUs) [27]. Mehta et al. demonstrated the efficacy of alcohol-based hand rubs in the disinfection of stethoscopes [28]. Therefore, hand rubs can be used for disinfection of stethoscopes and maintainence of hand hygiene. Educational and promotional campaigns for HCWs are required to achieve better compliance regarding stethoscope disinfection practices which can minimize HAIs [29].

### Implications for policy and practice

Based on the findings of this study, stethoscopes used by HCWs are contaminated with microorganisms. Hence, identification of microorganisms and to access their role as potential pathogens is mandatory as nosocomial pathogens may spread among patient’s and HCWs. Survey of stethoscope disinfection practices among HCWs is necessary because contamination and further spread of microorganisms is greatly reduced by regular cleaning stethoscope with suitable disinfectant. Performing AST is required to identify antibiotic resistant strains from as stethoscope used by HCWs as these can transfer among patients and HCWs. Motivating and training the HCWs regarding routine simple disinfection of stethoscope and regular maintainence of hand hygiene into practice can be an important step to reduce burden of nosocomial infections.

## Conclusions

Stethoscopes used by HCWs are contaminated with pathogenic microorganisms and therefore are potential vectors for the transmission of hospital pathogens. This contamination and spread of organisms can be greatly reduced by regular disinfection of stethoscope with 70% ethanol. There is a definite need for strict adherence to disinfection practices by HCWs to minimize cross-contamination and ensure patient safety in the hospital. Therefore, we need to train and motivate the HCWs in understanding different aspects of stethoscope disinfection practices. It could be an important step of intervention to minimize transmission of nosocomial pathogens among patients and HCWs.

## Limitations

Other contaminating organisms like anaerobic bacteria, fungi and viruses were not studied. The time period of contact of the stethoscope with the patient’s skin/clothes was not known. This study utilized only 70% ethanol we did not compare other alcohol and non-alcohol based products as a disinfecting agent. It is not known whether cleaning with alcohol will damage stethoscope diaphragms. Future research should focus on identification of other contaminating organisms and their role as nosocomial pathogens. More effective ways and practicable means of stethoscope disinfection.

## Additional file

**Additional file 1.** Questionnaire.

## Abbreviations

ABST: antibiotic sensitivity test
CFUs: colony-forming units
CMC: Chitwan medical college
CONS: coagulase negative staphylococci
HCAIs: health care associated infections
HCW_S_: health care workers
ICU: intensive care unit
MDR: multidrug resistant
MRSA: methicillin resistant *Staphylococcus aureus*
WHO: World Health Organization
