# Supplementary material for: Bacteriological assessment of stethoscopes used by healthcare workers in a tertiary care centre of Nepal
Source: BMC Res Notes. 2017 Jul 28;10:353. doi: 10.1186/s13104-017-2677-7 (PMC5534059; doi:10.1186/s13104-017-2677-7)
Supplement: Supplementary file 1 — Additional file 1. Questionnaire. [file 13104_2017_2677_MOESM1_ESM.docx]

**PROFORMA**

Name of the participant: …………………………………………………………………

Department/Ward: ……………………………………………………………………….

Date: ………………………………………………………………………………………

- Designation of Health Care Worker?

a. Consultants b. Medical Officer c. Medical intern d. Nurse

e. Post-graduate student

- Gender

1. Male b. Female

- Can Stethoscope transmit infectious agent?

a. Yes b. No

- Does it need to be disinfected?

a. Yes b. No

- When do you use stethoscope?

a. After removing clothes b. Without removing clothes c. Both

- Frequency of disinfection of stethoscopes.

1. Everyday b. Alternate day c. Once a week d. Once a month

e. >Once yearly f. Never cleaned

- Methods practiced for cleaning of stethoscopes?

a. Methylated Sprit swab b. Hand sanitizer c. Cloth

d. Soapy water e. No agent/Never Cleaned

- Hand washing procedure after seeing each patient?

a. Yes b. No

- Stethoscope cleaning after every patient

a. Yes b. No

- Barriers to cleaning of stethoscopes

a. Lack of time

b. Forgetfulness / Laziness

c. Lack of knowledge regarding best disinfectant

d. Lack of access to disinfectants

e. Concern for damaging one’s stethoscope

f. Sharing of stethoscopes

g. Unspecified
